# Screening for treatment-required sleep apnoea in patients with spinal cord injury within one year after injury in a rehabilitation setting

**DOI:** 10.1007/s11325-024-03062-9

**Published:** 2024-05-15

**Authors:** Erhard Trillingsgaard Næss-Schmidt, Anne Christensen, Jørgen Vibjerg, Vivi Lindhardt Hasselager, Louise Lindenmayer, Helle Susanne Laursen, Jørgen Feldbæk Nielsen, Filip Ivanov Kirov

**Affiliations:** 1 Hammel Neurorehabilitation Centre and University Clinic, Aarhus University, Aarhus, Denmark; 2 Department of Clinical Medicine, Faculty of Health, Aarhus University, Aarhus, Denmark; 3 Spinal Cord Injury Centre of Western Denmark, Regional Hospital of Viborg, Viborg, Denmark; 4 Department of Neurology, Regional Hospital of Viborg, Viborg, Denmark

**Keywords:** Stop Bang Questionnaire, Cardiorespiratory monitoring, Spinal cord injury, Rehabilitation, Sleep disorder, Sleep apnoea

## Abstract

**Purpose:**

The current study aims to assess the efficacy of the Stop-Bang Questionnaire (SBQ) in screening treatment-required sleep apnoea following Spinal Cord Injury (SCI). Additionally, we explore the performance of combined questionnaires and pulse oximetry to determine the most cost-effective method.

**Methods:**

The study employs a cross-sectional observational design. All patients admitted to in-hospital rehabilitation at the Spinal Cord Injury Centre of Western Denmark from September 2022 to February 2023 were continuously enrolled. Participating patients underwent SBQ screening, a standard sleep questionnaire, and cardiorespiratory monitoring, followed by an individual consultation with a physician.

**Results:**

During the study period, 35 SCI patients were admitted, with 24 providing informed consent. Among the 24 included patients, there was a 75% prevalence of mild to severe sleep apnoea, and 46% had treatment-required sleep apnoea. The SBQ missed only one patient with treatment-required sleep apnoea but misclassified eight patients. Combining SBQ with the pulse oximetry demonstrated the best performance in identifying patients with sleep apnoea.

**Conclusion:**

The study indicates that SBQ alone is insufficient for screening treatment-required sleep apnoea. Exploratory analysis suggests that combining SBQ with a simple pulse oximetry measurement might enhance accuracy.

## Introduction

Sleep apnoea is a prevalent sleep disorder among patients with spinal cord injury (SCI), exhibiting higher representation compared to the general population [1]. A systematic review focusing on sleep apnoea in chronic-phase tetraplegic patients reports a prevalence ranging from 46 to 97% [2]. Notably, increased prevalence is also observed among paraplegic patients [3]. Despite this, studies indicate that sleep apnoea in SCI patients is frequently underdiagnosed, emphasizing the necessity for systematic screening in both acute and community care settings [2, 4].

Obstructive Sleep Apnoea (OSA) is a sleep-related breathing disorder which is characterized by repetitive episodes of complete (apnoea) or partial (hypopnea) upper airway obstruction during sleep [5]. CSA is characterized by reduction or cessation of airflow due to reduced or absent respiratory effort [5].

There are several reasons to higher risk of OSA in both tetraplegic and paraplegic patients which include both physiological and neurological factors. First, the impairment in muscle function typical of tetraplegia can significantly affect the muscles that control breathing, including the diaphragm and intercostal muscles [4]. This muscle weakness can lead to reduced lung volumes and difficulty maintaining open airways during sleep. Additionally, the loss of normal neurological control over breathing during sleep further exacerbates the risk [2]. These complications are compounded by the higher likelihood of obesity and other secondary health conditions in both tetraplegic and paraplegic individuals [4], which can obstruct the upper airway and disrupt normal breathing patterns during sleep. Together, these factors contribute to the heightened prevalence of sleep apnea in this patient group, necessitating careful monitoring and management.

Untreated sleep apnea poses a significant risk of long-term adverse outcomes in the able-bodied population, particularly among men [6]. Moreover, there is growing evidence suggesting that untreated sleep apnoea in individuals with SCI may contribute to heightened neuropathic pain, increased spasticity, and cardiovascular autonomic dysfunction, all of which are currently subjects of ongoing research[7].

Assessment of sleep apnoea can be done with polysomnography (PSG), which is the golden standard, or ambulatory sleep apnoea monitoring like cardiorespiratory monitoring (CRM) [8]. Both examinations take time and require a team of professionals with expertise in sleep and are therefore costly to perform in all hospitalized patients. Therefore, there is a need for more efficient and cost-effective tools for screening sleep apnoea in patients with SCI.

Previous studies suggest predictors of OSA such as e.g. older age, snoring, body mass index (BMI) and male gender [9] which has been used in several questionnaires concerning OSA [9, 10]. Two meta-analysis examining questionnaires screening for OSA showed the Stop Bang Questionnaire (SBQ) to be the most accurate and sensitive screening questionnaire for detection of OSA in a mixed patient population [9, 10]. Opposite the SBQ show low specificity and therefore screening of sleep apnoea with SBQ may result in many false positives. A study by Graco et al. optimized screening of OSA in patients with tetraplegia using a two-step model. The model includes a questionnaire 'Screening for OSA in Tetraplegia' (SOSAT), similar to questions used in SBQ, combined with nocturnal pulse oximetry, which showed to increase specificity [11]. However, this model has not been investigated in a mixed population of tetra- and paraplegics. Moreover, SBQ performance has not yet been explored in a SCI population in a rehabilitation setting.

The purpose of the current study was to examine the performance of SBQ screening risk of treatment-required sleep apnoea (TRSA) in a para- and tetraplegic population of SCI within one year of injury in a rehabilitation setting. In addition, we explore the performance of combining SBQ, SOSAT and nocturnal pulse oximetry to see which method is most cost-effective for use of referral to further sleep examination.

## Method and materials

### Design

The study is a cross sectional observational study design and part of a quality project for assessment of sleep apnoea in a clinical setting.

### Setting

All patients admitted to in-hospital rehabilitation at Spinal Cord Injury Centre of Western Denmark (SCIWD) from September 2022 to February 2023 were continuously enrolled and assessed for sleep apnoea. SCIWD, one of two national centers in Denmark, specializes in providing highly specialized rehabilitation to patients with spinal cord-related injuries. Typically, patients admitted are in the sub-acute phase of their injury and are primarily referred from acute hospitals. SCIWD offers interdisciplinary rehabilitation and is affiliated with the Neurological department of the Regional Hospital Viborg, including a specialized sleep clinic at this location.

### Participants

All first-time hospitalized patients with SCI were consecutively invited to participate during the five-month period. The inclusion criteria were SCI (all causes) and registration as a Danish citizen. Patients were excluded if they had a tracheal tube, a previous diagnosis of sleep apnoea, inability to provide informed consent, a time since injury exceeding 12 months, or a lack of willingness to participate.

### Measurements

Participating patients underwent screening using the SBQ, a standard sleep questionnaire that includes the Epworth Sleepiness Scale (ESS) and a CRM examination, followed by a clinical assessment. The decision for TRSA was made by an experienced sleep physician based on the Clinical Guideline outlined by the American Academy of Sleep Medicine (AASM) [12]. According to the guideline, sleep apnoea is defined as mild when the Apnoea-Hypopnea Index (AHI) is 5–14 events per hour, moderate when 15–29 events per hour, and severe if there are more than 29 events per hour [8]. The diagnosis of TRSA was confirmed if the number of obstructive events exceeded 15 events per hour. Nevertheless, the final decision for TRSA was based on the comprehensive CRM assessment combined with the clinical evaluation. Mild sleep apnoea could also warrant treatment if complicated neurologic, heart, cerebrospinal or pulmonary diseases were present, severe isolated desaturations were observed, there were severe symptoms of sleepiness possibly related to sleep apnoea, or paroxysmal atrial fibrillation was present. According to these criteria, patients were categorised in four groups, 1: patients with TRSA, 2: patients with Mild to Moderate Sleep Apnoea (MTM-SA) without additional criteria for TRSA, 3: patients with No Sleep Apnoea (NO-SA) defined as AHI < 5, and 4: patients with No TRSA (NO-TRSA) including a combined group of MTM-SA and NO-TRSA.

#### CRM examination

The CRM examination involved portable monitor recordings using NOX-T3 by ResMed Maribo. The following signals were recorded: nasal pressure, rib cage and abdominal movement by inductance plethysmography, snoring, body position, activity, heart rate, and oxygen saturation by pulse oximetry to measure the Oxygen Desaturation Index (ODI). Apnoea's were defined as a ≥ 90% reduction in airflow from baseline for at least 10 s. Obstructive apnoea's were defined as apnoea's associated with respiratory effort, and central apnoea's were defined as apnoea's during which respiratory effort was absent. Mixed apnoea's were defined as apnoea's during which respiratory effort was initially absent but appeared during the latter part of the event. Hypopneas were defined as a ≥ 30% reduction in a respiratory signal for ≥ 10 s associated with a ≥ 4% reduction in oxygen saturation. ODI was graded into three groups: no oxygen desaturation (ODI < 5); mild (ODI 5–15) and moderate to severe (ODI ≥ 15). The recordings were manually scored by two experienced nurses.

#### SBQ

The SBQ, initially developed to assess the risk of OSA as a part of preoperative assessments for surgical patients [13], has since been utilized in various patient groups [9]. It comprises eight dichotomous (yes/no) items related to clinical features of OSA: loud snoring, tiredness, observed apnoea, high blood pressure, body mass index (BMI) (> 35 kg/m^2^), age (> 50 years), neck circumference (≥ 41 cm for female and ≥ 43 cm for male), and gender (male). Patients with a score of 3 or more are classified as having an intermediate risk of OSA. The questionnaire has been validated in a Danish population of patients referred to a sleep clinic [14]. The tool is sensitive in detecting moderate and severe OSA but is less effective in excluding mild or no OSA [15].

#### SOSAT

The SOSAT questionnaire, developed by Graco et al., consists of four items, using a threshold of ≥ 5 points out of 10, indicating sleep apnoea [11]. The SOSAT items include two variables with a weighting of 3 points (ASIA impairment scale (AIS) score A, B or C and self-reported snoring) and two variables with a weighting of 2 points (self-reported apnoea's and sleepiness). Three out of four SOSAT questions were adapted from the SBQ, and the AIS score was obtained from the clinical examination.

#### Sleep questionnaire

A standard clinical sleep questionnaire was utilized to support the clinical decision regarding sleep apnoea. The questionnaire included demographic data, co-morbidities, symptoms related to sleep quality, and single questions of e.g. daytime sleepiness and restless legs syndrome, all based on the Clinical Guideline produced by AASM [12]. Furthermore, the questionnaire contained the ESS for measurement of sleepiness [16].

### Data analysis

The screening for risk of TRSA was evaluated with four approaches. First, the performance of SBQ alone was examined using a threshold of ≥ 3 points for SBQ compared with the clinical decision of TRSA. Second, the performance of SOSAT alone was evaluated using a threshold of ≥ 5 points, as proposed by Graco et al. [11], compared with the clinical decision of TRSA. The third approach was a two-step approach, initially screening with SOSAT using the threshold of ≥ 5 points and subsequently screening positives of SOSAT with pulse oximetry using ODI ≥ 13, as proposed by Graco et al. [11] compared with the clinical decision of TRSA. The last approach was also a two-step approach, first screening with SBQ using a threshold of ≥ 3 points and then screening positives of SBQ with pulse oximetry using ODI ≥ 13 compared with the clinical decision of TRSA. Accuracy was calculated with the formula: true-positive (TP) + true-negative (TN) divided by TP + TN + false-positive + false-negative.

### Statistics

Continuous variables are presented as mean ± standard deviation. For non-normally distributed distributions, median, interquartile range, and minimum and maximum values are provided. Prevalence is reported with 95% confidence interval. Agreements are depicted with 2 × 2 cross tables, and performance metrics, including sensitivity, specificity, and accuracy, are reported. Statistical analyses were conducted using STATA 17.0 (25).

## Results

A total of 35 patients with SCI were admitted to SCIWD from September 2022 to February 2023. Of these, 24 provided informed consent and underwent examination with CRM; demographic details are presented in Table 1. Eleven patients were not included in the study. One patient had a tracheal tube, three were already diagnosed with sleep apnoea, two were unable to give informed consent and five patients declined to participate. No patients were excluded solely to TSI > one year.

**Table 1:** Baseline characteristics

	Included, n = 24	Not included, n = 11
Age (years), median (range)	53 (26—76)	66 (23—81)
Gender	6	5
Female, n	18	6
Male, n		
BMI_a_ (kg/m^2^), median (range)	26.9 (21.1—39.2)	22.5 (20.0—29.9)
TSI_b_ (months), median (range)	2.4 (0.4—8.1)	5.4 (1.2—446)
Level of injury, n		
Tetraplegia	13	8
Paraplegia	11	3
Severity (ASIA_c_ Index Scale), n		
A	0	2
B	0	1
C	2	0
D	22	7
Missing	0	1
ESS_d_, n		
0 to 10 (normal range)	20	1
11 to 14 (mild sleepiness)	3	1
15 to 17 (moderate sleepiness)	1	0
18 to 24 (severe sleepiness)	0	0
missing	0	9

a = Body Mass Index, b = Time Since Injury, c = American Spinal Injury Association, d = Epworth Sleepiness Scale

Among the 24 included patients, there was a prevalence of sleep apnoea of 75% (CI 853.3–90.2), based on AHI > 5%, and a prevalence of 45.8% (CI 25.6–67.2) of patients with TRSA. Except for one patient, both AHI ≥ 15 and ODI ≥ 15 were discriminators of TRSA versus MTM-SA and NO-SA. Moreover, the supine sleeping position and blood pressure within non-dipper range were more prevalent in the TRSA group. Data from the CRM examination is presented in Table 2.

**Table 2:** Cardiorespiratory measurements

	TRSA_a_, n = 11	MTM-SA_b_, n = 7	NO-SA_c_, n = 6
AHI_d_, n			
AHI < 5	0	0	6
AHI 5 to 15	1	7	0
AHI 15 to 29	7	0	0
AHI ≥ 30	3	0	0
ODI_e_, n			
ODI < 5	1	0	2
ODI 5 to 15	0	7	4
ODI ≥ 15	9	0	0
Missing, n	1	0	0
Respiration rate/min, median (range)	13.2 (10.1—18)	16.0 (13.2—21.1)	14.8 (2.8)
Central Apnoea-hypopnea/hour, median (range)	0.4 (0—6.7)	0.6 (0.1—2.1)	0.1 (0—0.4)
Minimum SpO_2_ percentage, median (range)	78 (0—86)	87 (77—89)	81 (77—92)
Missing, n	1	0	0
Supine sleeping position (%), median (range)	75.5 (13.1—100)	61.1 (1.5—100)	13.0 (1—60)
Within non-dipper range, n	7	4	1
Missing, n	1	1	2

a = Treatment-Required Sleep Apnoea, b = Mild To Moderate Sleep Apnoea, c = No Sleep Apnoea, d = Apnoea-Hypopnea Index, e = Oxygen Desaturation Index

All items of the SBQ predicting the risk of sleep apnoea were relatively more represented in the TRSA group. Responses to specific SBQ items in patients with TRSA versus NO-TRSA are presented in Table 3.

**Table 3:** Stop Bang Questionnaire results

	TRSA_a_, n = 11, n (%)	NO-TRSA_b_, n = 13, n (%)
Snoring	8 (73)	6 (46)
Tired	7 (64)	3 (23)
Observed apnoea	3 (27)	2 (15)
Treatment for hypertension	4 (36)	4 (31)
BMI_c_ > 35 kg/m^2^	4 (36)	0 (0)
Age > 50	9 (82)	6 (46)
Neck circumference ≥ 41/43	7 (64)	4 (31)
Male	10 (91)	8 (62)

a = Treatment-Required Sleep Apnoea, b = NO Treatment-Required Sleep Apnoea, c = Body Mass Index

The SBQ missed only one patient with TRSA but misclassified eight patients with TRSA who did not have TRSA. The agreement between TRSA and NO-TRSA determined with CRM versus the SBQ is presented in Table 4.

**Table 4:** Agreement between treatment-required sleep apnoea and Stop Bang Questionnaire

	TRSA_a_, n = 11, n	NO-TRSA_b_, n = 13, n	Total
Positive SBQ_c_	10	8	18
Negative SBQ_c_	1	5	6
Total	11	13	24

a = Treatment-Required Sleep Apnoea, b = NO Treatment-Required Sleep Apnoea, c = Stop Bang Questionnaire

The SBQ and ODI combination constituted the model with the highest sensitivity, specificity, and accuracy for identifying patients with TRSA. Screening with SBQ alone resulted in the highest number of referrals to CRM and pulse oximetry. When SBQ was combined with pulse oximetry, it missed three patients with TRSA, classified eight with TRSA, and demonstrated the best overall performance. The sensitivity, specificity, and accuracy of the four approaches, along with the number of patients referred to ODI and CRM after initial screening with SBQ and SOSAT, are presented in Table 5.

**Table 5:** Sensitivity, specificity, accuracy, and referral to oximetry and/or cardiorespiratory monitoring for the four approaches

n = 23	Sensitivity, % (95% CI)	Specificity, % (95% CI)	Accuracy, %	Referral to ODI_c_, n (%)	Referral to CRM_d_, n (%)
SBQ_a_	90.0 (55.5—99.7)	38.5 (13.9—68.4)	60.1	-	18 (78%)
SOSAT_b_	60.0 (26.2—87.8)	84.6 (54.6—98.1)	73.9	-	8 (35%)
SOSAT and ODI_c_	60.0 (26.2—87.8)	100 (75.3—100)	82.6	8 (35%)	6 (26%)
SBQ and ODI	80.0 (44.45—97.5)	100 (75.3—100)	91.3	18 (78%)	8 (35%)

a = Stop Bang Questionnaire, b = Screening for Obstructive Sleep Apnoea in Tetraplegia, c = Oxygen Desaturation Index, d = CardioRespiratory Monitoring

## Discussion

This study represents the first exploration of the SBQ performance within a mixed population of tetra- and paraplegic individuals with SCI within one year of injury, specifically in a rehabilitation setting. The findings of this investigation reveal that while SBQ demonstrates sensitivity in detecting TRSA, it lacks specificity. Consequently, relying solely on SBQ proves ineffective in screening for the risk of sleep apnoea, as it results in numerous additional examinations, such as CRM, to identify patients with TRSA. The secondary outcomes from all four approaches propose that a more effective approach involves combining SBQ with pulse oximetry. This combined approach not only enhances specificity and sensitivity but also strikes a balance in identifying patients at risk of sleep apnoea. Importantly, it reduces unnecessary referrals for CRM examinations, thereby optimizing the efficiency of the screening process.

The SBQ performance in the current study is in alignment with previous studies in other populations [9]. A study of SBQ validation in patients with multiple sclerosis showed that the SBQ was an efficient screening tool for moderate and severe sleep apnoea but not sensitive enough to exclude mild sleep apnoea [15]. Therefore, the finding of SBQ being sensitive and not specific in the current study may not seem surprisingly. However, when adding ODI, as suggested by Graco et al., both sensitivity and specificity increased. Moreover, using SBQ instead of SOSAT combined with ODI made the screening even more accurate. There may be several explanations for this result. First, the population in the Graco study were tetraplegics with more severity than patients in the current study. Although the SBQ and the SOSAT are similar the SOSAT was developed to be sensitive in a tetraplegic population. In our study only two of the participants had an AIS score of C and the rest were classified D. Therefore, most of the participants did not get three points for AIS A, B or C in the SOSAT questionnaire which reduced the total SOSAT score and classification of TRSA. This finding suggests the existence of a subgroup of less severe tetraplegic patients who still suffer from sleep apnea but are not detected by the SOSAT. Opposite, the SBQ alone classified eight false positive patients with TRSA which would be referred to CRM assessment and thus being ineffective in referral of the right patients. It has previously been suggested that cervical injury level [17] and severity [18] predict higher risk of sleep apnoea. However, these two variables are likely to reduce the sensitivity for detection of sleep apnoea in a mixed SCI population of para- and tetraplegia as seen in our results from the SOSAT performance regarding to no points for severity of AIS D and might need another weighting. Intriguingly, another compelling variable to consider is the measurement of blood pressure, specifically patients within the non-dipper range. This becomes particularly significant in the TRSA patients, where the prevalence of non-dipper patterns is notably higher [19]. Although the dynamic interplay between hypertension, nocturnal dips, and their direct connection to sleep apnea remains a subject of speculation [20], a larger validation study exploring relevant variables would be interesting and needed to answer if there is a better balance of questions and objective items to catch patient with TRSA. On the other hand, adding the ODI examination already seems both feasible and demand little resources and the proposed model of SBQ and ODI may be cost-effective enough for use of referral to CRM or PSG although it does miss a few subjects.

It is well known that sleep apnoea are highly prevalent in the able-bodied population [21], but the results in the current study and previous studies [3, 4] point toward that the population of both para- and tetraplegics are in higher risk. Moreover, two longitudinal studies point toward a relatively stable condition of sleep apnoea in both para- and tetraplegics from acute up to one year after injury [22, 23]. Therefore, the condition of sleep apnoea does not seem to change from acute to chronic and warrant action in the initial phase to prevent co-morbidity associated with sleep apnoea [18]. Recently reviews of sleep disturbance after SCI suggest that several physiologic conditions of e.g. heart rate, blood pressure, energy expenditure, sleeping metabolic rate and bowel movements may be altered [4, 18]. Some of these conditions may also have an impact from untreated sleep apnoea and therefore a special interest to address sleep apnoea in patients with SCI and adding additional tools to become more accurate to find those in need of treatment are wanted [4, 24].

There are several limitations to the study. First, the small sample size is a limitation, and the results demand further validation. However, the results from both SBQ alone and ODI in combination are in line with other studies [9, 11]. Furthermore, patient reported outcome on e.g. snoring and apnoea might be prone to reporting bias which could underestimate the risk of sleep apnoea and call for more objective methods. Another possibility of bias is related to the Clinical decision of TRSA. It is well known that PSG is the golden standard for sleep studies. CRM is a well approved method for diagnosing sleep apnoea but may overestimate the group in risk [25]. However, the manual scoring of CRM as performed in the current study has previous shown that CRM is almost as good as PSG in classifying TRSA [25]. Furthermore, we assume that the thorough examination of an experienced physicians in addition to CRM captured the most important patients in need of treatment. However, the CRM and use of ODI and simply questions cannot stand alone and may only be the initial screening to refer more relevant patient to a full PSG examination. Finally, the used setup of the SOSAT questionnaire was not identical to the original questionnaire. Three similar items from SBQ were used to replace items from SOSAT in addition to the AIS score. However, although the questions were almost alike, and we assume that the response to the questions would be the same, this requires further validation.

This is the first study to report on the SBQ as a screening tool for sleep apnoea in an early rehabilitation setting of patients with SCI. Our study shows that SBQ alone might not be applicable to patients with SCI as a screening tool for TRSA. Furthermore, our explorative analysis shows that the SBQ in combination with a simple pulse oximetry measurement might be more accurate. The current results warrant validation in a larger sample size to find out if SBQ in combination with pulse oximetry is a reliable and valid method to screen for TRSA in mixed tetra-/paraplegic population of SCI.

## Data Availability

The data that support the findings of this study are not openly available due to reasons of sensitivity and are available from the corresponding author upon reasonable request. Data are located in controlled access data storage (REDCap) in The Central Denmark Region.

## References

[1] Ding W et al (2022) Spinal cord injury: the global incidence, prevalence, and disability from the global burden of disease study 2019. Spine (Phila Pa 1976) 47(21):1532–1540 35857624 10.1097/BRS.0000000000004417 PMC9554757

[2] Graco M et al (2021) Prevalence of sleep-disordered breathing in people with tetraplegia-a systematic review and meta-analysis. Spinal Cord 59(5):474–484 33446931 10.1038/s41393-020-00595-0

[3] Chiodo AE, Sitrin RG, Bauman KA (2016) Sleep disordered breathing in spinal cord injury: A systematic review. J Spinal Cord Med 39(4):374–382 27077573 10.1080/10790268.2015.1126449 PMC5102283

[4] Hultén VDT et al (2020) A review of sleep research in patients with spinal cord injury. J Spinal Cord Med 43(6):775–796 30513274 10.1080/10790268.2018.1543925 PMC7808257

[5] Sateia MJ (2014) International classification of sleep disorders-third edition: highlights and modifications. Chest 146(5):1387–1394 25367475 10.1378/chest.14-0970

[6] Kendzerska T et al (2014) Untreated obstructive sleep apnea and the risk for serious long-term adverse outcomes: a systematic review. Sleep Med Rev 18(1):49–59 23642349 10.1016/j.smrv.2013.01.003

[7] Furlan JC, Loh E, Boulos MI (2023) The potential effects of untreated sleep-related breathing disorders on neuropathic pain, spasticity, and cardiovascular dysfunction following spinal cord injury: A cross-sectional prospective study protocol. PLoS ONE 18(5):e0282860 37130111 10.1371/journal.pone.0282860 PMC10153696

[8] Kapur VK et al (2017) Clinical practice guideline for diagnostic testing for adult obstructive sleep apnea: an american academy of sleep medicine clinical practice guideline. J Clin Sleep Med 13(3):479–504 28162150 10.5664/jcsm.6506 PMC5337595

[9] Chiu HY et al (2017) Diagnostic accuracy of the Berlin questionnaire, STOP-BANG, STOP, and Epworth sleepiness scale in detecting obstructive sleep apnea: A bivariate meta-analysis. Sleep Med Rev 36:57–70 27919588 10.1016/j.smrv.2016.10.004

[10] Amra B et al (2018) Screening questionnaires for obstructive sleep apnea: an updated systematic review. Oman Med J 33(3):184–192 29896325 10.5001/omj.2018.36 PMC5971053

[11] Graco M et al (2018) Diagnostic accuracy of a two-stage model for detecting obstructive sleep apnoea in chronic tetraplegia. Thorax 73(9):864–871 29735608 10.1136/thoraxjnl-2017-211131

[12] Epstein LJ et al (2009) Clinical guideline for the evaluation, management and long-term care of obstructive sleep apnea in adults. J Clin Sleep Med 5(3):263–276 19960649 10.5664/jcsm.27497 PMC2699173

[13] Chung F et al (2008) STOP questionnaire: a tool to screen patients for obstructive sleep apnea. Anesthesiology 108(5):812–821 18431116 10.1097/ALN.0b013e31816d83e4

[14] Bille J, Bille-Hasselstrøm C, Petersen CG (2015) Translation and validation of the Stop-Bang Questionnaire for obstructive sleep apnoea into Danish. Danish Medical J 62(12):A5158 26621392

[15] Singh M et al (2022) Validation of an obstructive sleep apnea symptom inventory in persons with multiple sclerosis. Mult Scler 28(2):280–288 34048308 10.1177/13524585211013014 PMC8627523

[16] Johns MW (1991) A new method for measuring daytime sleepiness: the Epworth sleepiness scale. Sleep 14(6):540–545 1798888 10.1093/sleep/14.6.540

[17] Sankari A et al (2014) Sleep disordered breathing in chronic spinal cord injury. J Clin Sleep Med 10(1):65–72 24426822 10.5664/jcsm.3362 PMC3869071

[18] Squair JW et al (2019) Sleep-disordered breathing is associated with brain vascular reactivity in spinal cord injury. Neurology 93(24):e2181–e2191 31694923 10.1212/WNL.0000000000008619 PMC6937492

[19] Friedman O, Logan AG (2009) Nocturnal blood pressure profiles among normotensive, controlled hypertensive and refractory hypertensive subjects. Can J Cardiol 25(9):e312–e316 19746250 10.1016/S0828-282X(09)70142-4 PMC2780906

[20] Phillips CL, O’Driscoll DM (2013) Hypertension and obstructive sleep apnea. Nat Sci Sleep 5:43–52 23750107 10.2147/NSS.S34841 PMC3666153

[21] Hang LW et al (2015) Validation of overnight oximetry to diagnose patients with moderate to severe obstructive sleep apnea. BMC Pulm Med 15:24 25880649 10.1186/s12890-015-0017-z PMC4407425

[22] Berlowitz DJ et al (2005) A longitudinal evaluation of sleep and breathing in the first year after cervical spinal cord injury. Arch Phys Med Rehabil 86(6):1193–1199 15954059 10.1016/j.apmr.2004.11.033

[23] Bauman KA et al (2016) Simplified Approach to Diagnosing Sleep-Disordered Breathing and Nocturnal Hypercapnia in Individuals With Spinal Cord Injury. Arch Phys Med Rehabil 97(3):363–371 26297810 10.1016/j.apmr.2015.07.026

[24] Adler D, Janssens JP (2018) Estimating the probability of OSA in the spinal cord injury population: specific tools are still needed. Thorax 73(9):803–805 29921701 10.1136/thoraxjnl-2018-211954

[25] Lux L, Boehlecke B, Lohr KN (2004) In effectiveness of portable monitoring devices for diagnosing obstructive sleep apnea: update of a systematic review. Rockville (MD) 26065047

